# Extended phase graph formalism for systems with magnetization transfer and exchange

**DOI:** 10.1002/mrm.27040

**Published:** 2017-12-15

**Authors:** Shaihan J. Malik, Rui Pedro A.G. Teixeira, Joseph V. Hajnal

**Affiliations:** ^1^ Department of Biomedical Engineering School of Biomedical Engineering and Imaging Sciences, King's College London, St. Thomas' Hospital London, SE1 7EH United Kingdom; ^2^ Centre for the Developing Brain School of Biomedical Engineering and Imaging Sciences, King's College London, St. Thomas' Hospital London, SE1 7EH United Kingdom

**Keywords:** extended phase graphs, Bloch‐McConnell equations, magnetization transfer, sequence simulation, exchange, relaxometry

## Abstract

**Purpose:**

An extended phase graph framework (EPG‐X) for modeling systems with exchange or magnetization transfer (MT) is proposed.

**Theory:**

EPG‐X models coupled two‐compartment systems by describing each compartment with separate phase graphs that exchange during evolution periods. There are two variants: EPG‐X(BM) for systems governed by the Bloch‐McConnell equations, and EPG‐X(MT) for the pulsed MT formalism. For the MT case, the “bound” protons have no transverse components, so their phase graph consists of only longitudinal states.

**Methods:**

The EPG‐X model was validated against steady‐state solutions and isochromat‐based simulation of gradient‐echo sequences. Three additional test cases were investigated: (i) MT effects in multislice turbo spin‐echo; (ii) variable flip angle gradient‐echo imaging of the type used for MR fingerprinting; and (iii) water exchange in multi‐echo spin‐echo T_2_ relaxometry.

**Results:**

EPG‐X was validated successfully against isochromat based transient simulations and known steady‐state solutions. EPG‐X(MT) simulations matched in‐vivo measurements of signal attenuation in white matter in multislice turbo spin‐echo images. Magnetic resonance fingerprinting–style experiments with a bovine serum albumin (MT) phantom showed that the data were not consistent with a single‐pool model, but EPG‐X(MT) could be used to fit the data well. The EPG‐X(BM) simulations of multi‐echo spin‐echo T_2_ relaxometry suggest that exchange could lead to an underestimation of the myelin‐water fraction.

**Conclusions:**

The EPG‐X framework can be used for modeling both steady‐state and transient signal response of systems exhibiting exchange or MT. This may be particularly beneficial for relaxometry approaches that rely on characterizing transient rather than steady‐state sequences. Magn Reson Med 80:767–779, 2018. © 2017 The Authors Magnetic Resonance in Medicine published by Wiley Periodicals, Inc. on behalf of International Society for Magnetic Resonance in Medicine. This is an open access article under the terms of the Creative Commons Attribution License, which permits use, distribution and reproduction in any medium, provided the original work is properly cited.

## INTRODUCTION

The extended phase graph (EPG) algorithm 1, 2, 3 is a commonly used tool for simulating signals obtained from MRI pulse sequences including multiple radiofrequency (RF) and gradient pulses, both qualitatively and quantitatively. It has been used for a diverse and growing range of applications including characterization of RF spoiling in gradient‐echo sequences 4, 5, analysis of echo amplitudes in turbo spin‐echo (TSE) sequences 6, 7, 8, 9, parallel transmission sequence design 10, 11, diffusion effects 12, and characterizing signal evolution in sequences used for relaxometry 13, 14, 15, 16.

The EPG method is a Fourier approach to solving the Bloch equations, and therefore assumes that tissues are characterized by a single set of relaxation parameters. It is recognized, though, that a single‐compartment approach fails to fully characterize complex biological tissues in many circumstances. Instead, coupled multicompartment models have been proposed. The Bloch‐McConnell (BM) equations 17 are a general form for describing systems that are coupled via a general exchange process, with a modification to further describe magnetization transfer 18, 19. The MT effects in particular have been shown to be strong determinants of observed signals in human tissue (e.g., ref. 20 shows that on‐resonance MT effects are expected to change the signal from balanced steady‐state free‐precision (SSFP) sequences in brain by approximately 30% and muscle by 50%).

The EPG formalism provides a computationally efficient method for the modeling of MR sequences that also gives intuitive insight into signal formation, by isolating different pathways that can lead to echo formation. Currently, it is not possible to use EPGs to model multicompartment systems with exchange (different compartments in nonexchanging systems can simply be modeled separately and then averaged 16). Hence, this work seeks to extend the EPG formalism to model such systems. This is increasingly relevant to emerging transient phase relaxometry approaches such as magnetic resonance fingerprinting (MRF) 21, which require numerical simulation, as opposed to more traditional steady‐state methods for which analytic or closed‐form solutions are often available (e.g., DESPOT or multicomponent DESPOT methods 22).

We first outline the proposed “EPG‐X” method, and then validate it against isochromat‐based simulations and established steady‐state solutions for gradient‐echo imaging. The EPG‐X calculations are then used to explore some test cases to illustrate the effect of the new approach. Test cases include multislice versus single‐slice TSE, and two different relaxometry methods: multi‐echo Carr‐Purcell‐Meiboom‐Gill (CPMG) data for multicomponent T_2_ estimation, and gradient‐echo imaging with modulated flip angles (similar to MRF). Experimental data were collected, both in vivo and on phantoms.

## THEORY

For simplicity, we consider in this work sequences with equidistant timing and an unbalanced gradient in a single direction. In this case, the intravoxel magnetization distribution resulting from a sequence of RF and gradient pulses may be characterized by the gradient‐induced phase ψ during some fixed time period Δt. An idealized voxel is defined by the interval ψ∈[−π,π] with uniform density of magnetization in this range. In the EPG representation the magnetization is represented by configuration states ${\tilde{F}}_{n}$ and ${\tilde{Z}}_{n}$, which, using the notation from the introductory review from Weigel (3), are defined as

(1) $$\begin{array}{l} M_{+}\left(\psi \right)=M_{x}\left(\psi \right)+iM_{y}\left(\psi \right)=\overset{\infty }{\underset{n=-\infty }{\sum }}{\tilde{F}}_{n}e^{in\psi }\\ M_{\_}\left(\psi \right)=M_{x}\left(\psi \right)-iM_{y}\left(\psi \right)=\overset{\infty }{\underset{n=-\infty }{\sum }}{\tilde{F}}_{-n}^{*}e^{in\psi }\\ M_{z}\left(\psi \right)=\overset{\infty }{\underset{n=-\infty }{\sum }}{\tilde{Z}}_{n}e^{in\psi } \end{array}$$

The signal at any time is the voxel average of $M_{+}$ (i.e., the value of ${\tilde{F}}_{0}$).

### Extension to Two Compartments

Consider a two‐compartment system, arbitrarily labeled as *a* and *b* with thermal equilibrium magnetizations $M_{0}^{a}=\left(1-f\right)M_{0}$ and $M_{0}^{b}=fM_{0}$, respectively. M_0_ is the total magnetization and *f* is the fraction in compartment *b*, which is conventionally assumed to be smaller. The BM equations governing evolution of this system in the absence of RF pulses may be written as

(2) $${\dot{M}}_{T}=\left(\Lambda_{T}+\Omega \right)\;M_{T}\;\;{\dot{M}}_{L}=\;\Lambda_{L}M_{L}+C.$$

where $M_{T}={\left[M_{+}^{a}\;M_{-}^{a}\;\;M_{+}^{b}\;M_{-}^{b}\right]}^{T}$ and $M_{L}={\left[M_{z}^{a}\;\;M_{z}^{b}\right]}^{T}\;$ correspond to transverse and longitudinal magnetization in each compartment. The other matrices are

(3a) $$\Lambda_{T}=\left[\begin{array}{cccc} -R_{2,a}-k_{a} & 0 & k_{b} & 0\\ 0 & -R_{2,a}-k_{a} & 0 & k_{b}\\ k_{a} & 0 & -R_{2,b}-k_{b}-2\pi i\delta_{b} & 0\\ 0 & k_{a} & 0 & -R_{2,b}-k_{b}+2\pi i\delta_{b} \end{array}\right]$$

(3b) $$\Lambda_{L}=\left[\begin{array}{cc} -R_{1,a}-k_{a} & k_{b}\\ k_{a} & -R_{1,b}-k_{b} \end{array}\right]$$

(3c) $$\Omega =\left[\begin{array}{cccc} -i\omega_{z} & 0 & 0 & 0\\ 0 & i\omega_{z} & 0 & 0\\ 0 & 0 & -i\omega_{z} & 0\\ 0 & 0 & 0 & i\omega_{z} \end{array}\right]$$

(3d) $$C={\left[R_{1,a}M_{0}^{a}\;R_{1,b}M_{0}^{b}\right]}^{T}$$

In these expressions, k_a_ is the exchange rate from compartment *a* to *b*, which is related to the reverse exchange rate k_b_ via $k_{a}M_{0}^{a}=k_{b}M_{0}^{b}$ to preserve balance at thermal equilibrium. R_1,a_ is the longitudinal relaxation rate for compartment *a* (i.e., 1/T_1,a_); and $\delta_{b}$ is the frequency offset (Hz) for compartment *b* relative to compartment *a*.

An EPG description of the two‐compartment model must include “states” that correspond to magnetization from *a* and *b*. Taking the Fourier transforms of Equation (2) and writing in terms of $F_{n}={\left[{\tilde{F}}_{n}^{a}\;{\tilde{F}}_{-n}^{*a}\;\;{\tilde{F}}_{n}^{b}\;{\tilde{F}}_{-n}^{*b}\;\right]}^{T}$ and $Z_{n}={\left[{\tilde{Z}}_{n}^{a}\;{\tilde{Z}}_{n}^{b}\right]}^{T}$, we obtain

(4) $${\dot{F}}_{n}=\left(\Lambda_{T}+\Omega \right)\;F_{n}$$

(5) $${\dot{Z}}_{n}=\;\Lambda_{L}Z_{n}+C\;\delta \left(n\right)$$

where superscripts *a* and *b* indicate the compartment, and it is understood that the full expression for the intravoxel magnetization distribution consists of sums over *n* as in Equation (1).

The solution to Equation (4) is $F_{n}\left(t+\Delta t\right)=\exp\left(\left(\Lambda_{T}+\Omega \right)\Delta t\right)\;F_{n}\left(t\right)$. We may take advantage of the fact that matrices $\Lambda_{T}$ and Ω  commute to re‐express the matrix exponential as a product of terms $\exp\left(\Lambda_{T}\Delta t\right)\exp\left(\Omega \Delta t\right)$ and define operators as

(6) Ψ≡exp(ΩΔt)

(7) $$\Xi_{T}\equiv \exp\left(\Lambda_{T}\Delta t\right)$$

such that $F_{n}\left(t+\Delta t\right)=\Psi \Xi_{T}F_{n}\left(t\right)$. Defining the dephasing during time Δt as $\psi =-\omega_{z}\Delta t$, we identify Ψ as the familiar “shift” operator, which increments the index of transverse states as with the standard EPG algorithm.

Equation (5) for $Z_{n}$ is homogeneous for *n* ≠ 0, but inhomogeneous for *n* = 0. The solutions for the two regimes are

(8) $$Z_{n}\left(t+\Delta t\right)=\Xi_{L}\;Z_{n}\left(t\right)\;(n\neq 0)$$

(9) $$Z_{0}\left(t+\Delta t\right)=\Xi_{L}\;Z_{0}\left(t\right)+(\Xi_{L}-I)\Lambda_{L}^{-1}C\;\left(n=\;0\right)$$

(10) $$\Xi_{L}\equiv \exp\left(\Lambda_{L}\Delta t\right)$$

This is in accordance with the standard EPG framework in which longitudinal recovery occurs only in the *n* = 0 state. The relaxation‐exchange operators $\Xi_{T}$ and $\Xi_{L}$ replace the relaxation operators from the standard EPG approach. Relaxation and exchange are treated simultaneously using these combined operators; it is not generally possible to separate these processes, because the corresponding components of the Λ matrices do not commute. The operators do not have a simple analytic form; instead, the matrix exponentials are evaluated numerically. The form of the Λ matrices means that exchange couples only states of the same “type” and “order” (e.g., ${\tilde{F}}_{n}^{a}↔{\tilde{F}}_{n}^{b}$ and ${\tilde{Z}}_{n}^{a}↔{\tilde{Z}}_{n}^{b}$).

### Solution for RF Pulses

In the classic EPG framework, RF pulses mix together all states for a given order *n* via transition operator (3) as follows:

(11) $$T_{\alpha \phi }=\left[\begin{array}{ccc} cos^{2}\frac{\alpha }{2} & e^{2i\phi }sin^{2}\frac{\alpha }{2} & -ie^{i\phi }sin\;\alpha \\ e^{-2i\phi }sin^{2}\frac{\alpha }{2} & cos^{2}\frac{\alpha }{2} & ie^{-i\phi }sin\;\alpha \\ -\frac{i}{2}e^{-i\phi }sin\;\alpha & \frac{i}{2}e^{i\phi }sin\;\alpha & cos\;\alpha \end{array}\right]$$

where α and ϕ are the RF pulse flip angle and phase. In the two‐compartment case, the overall transition matrix to apply to the full system ${\left[{\tilde{F}}_{n}^{a}\;{\tilde{F}}_{-n}^{*a}\;{\tilde{Z}}_{n}^{a}\;{\tilde{F}}_{n}^{b}\;{\tilde{F}}_{-n}^{*b}\;{\tilde{Z}}_{n}^{b}\right]}^{T}$ is simply

(12) $$T=\;[\begin{array}{cc} T_{\alpha_{a}\phi_{a}} & 0\\ 0 & T_{\alpha_{b}\phi_{b}} \end{array}].$$

where $\alpha_{a}$ is the flip angle for compartment *a*, and so on. This paper only considers scenarios in which the flip angle and phase are the same for both compartments. For situations in which $\delta_{b}$ is large compared with the RF pulse bandwidth, these could be different.

### Magnetization Transfer

A different formulation is generally used when describing MT effects in tissues with a “semisolid” component 18. In this case, compartment *b* is often referred to as the “bound” or “restricted” pool and is assumed to represent highly immobile protons whose T_2_ is very short (in the order of 10 µs). In this case we assume that compartment *b* has no transverse magnetization; hence, states ${\tilde{F}}_{n}^{b}\;$ and $\;{\tilde{F}}_{-n}^{*b}$ are dropped such that $F_{n}={\left[{\tilde{F}}_{n}^{a}\;{\tilde{F}}_{-n}^{*a}\right]}^{T}$. In this formulation, $F_{n}$ are treated exactly as in the classic EPG case (i.e., subject to T_2_ relaxation and shifts caused by gradients), and $\delta_{b}$ is not defined. The coupled longitudinal states $Z_{n}$ still evolve as per Equations (8) and (9).

The effect of RF pulses on compartment *a* is to rotate the magnetization as previously described. However, for compartment *b* (the “bound pool”), RF pulses act so as to directly saturate the longitudinal component with saturation rate $\bar{W\left(\omega_{z}\right)}$, which for pulsed saturation is defined as 19

(13) $$\bar{W\left(\omega_{z}\right)}=\frac{\pi \gamma^{2}}{\tau_{rf}}\overset{\tau_{rf}}{\underset{0}{\int }}B_{1}^{2}\left(t\right)dt\;G\left(\omega_{z}\right)$$

where B_1_(t) is the RF pulse waveform and $\tau_{rf}\;$ is its duration. This is a function of off‐resonance frequency $\omega_{z}$, because it depends on the absorption lineshape $G(\omega_{z})$. Different candidate lineshapes have been proposed for modeling semisolids in biological tissues with Gaussian 19 and super‐Lorentzian shapes 23 used primarily. The overall RF transition matrix is therefore

(14) $$T=\;[\begin{array}{cc} T_{\alpha \phi } & 0\\ 0 & e^{-\bar{W\left(\omega_{z}\right)}\tau_{rf}} \end{array}].$$

### Summary of Proposed Theory

**Figure 1:**
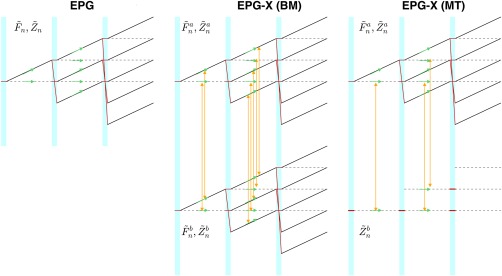
Left: Overview of single‐compartment extended phase graph (EPG) algorithm. ${\tilde{Z}}_{n}^{}$ states are represented by dotted lines, ${\tilde{F}}_{n}^{}$ by solid black lines. Blue‐shaded regions correspond to application of radiofrequency (RF) pulses. Red lines trace how states are mixed by the action of RF pulses. Green arrows depict relaxation effects, which occur individually to each state during the period between RF pulses. Middle: The EPG‐X (Bloch‐McConnell) approach consists of two separate EPGs. The RF pulses have the same effect as EPG. During evolution periods, relaxation (green arrows) and exchange (yellow arrows) both occur. Exchange links each state to its equivalent in the other compartment (i.e., ${\tilde{F}}_{n}^{a}↔{\tilde{F}}_{n}^{b}$). Right: The EPG‐X magnetization transfer (MT) model has a reduced second compartment represented only by ${\tilde{Z}}_{n}^{b}.$ The RF pulses directly saturate these states (Eq. (14)), and they exchange directly with their equivalents in compartment *a*. For clarity, in all diagrams, relaxation and exchange effects are only depicted for the periods following the first two RF pulses. Although depicted by separate arrows, relaxation and exchange processes are governed by single combined operations.

To summarize, we have introduced extensions to the EPG formalism to account for multicompartment systems with exchange (EPG‐X). There are two variants: one for systems governed by the BM equations, and one for the variant of BM often used for MT, in which one compartment has negligible transverse magnetization. Both effectively use two coupled EPG calculations: one for each compartment, although the MT variant uses a second compartment with longitudinal components only. These are summarized diagrammatically in Figure 1.

## METHODS

The EPG‐X framework could in principle be used to simulate the response of a two‐compartment system for any pulse sequence. The theory is illustrated by simulating four separate scenarios. The model tissue parameters used are outlined in Table 1; the subset used in each simulation is indicated in the text. In all models, compartment *b* is the smaller one. The myelin‐water exchange model is motivated by literature on exchange between water trapped within myelin layers (compartment *b*) and intra‐ and extra‐axonal water 24, but uses rounded values rather than taking numbers directly from a single source. In this model the myelin‐water mean residence time ( $\tau_{b})$ is given by $\tau_{b}=f/\left(k_{a}\left(1-f\right)\right)$ 22 (the parameters in Table 1 make $\tau_{b}\;$ = 100 ms consistent with 22, although estimates vary significantly) 24. There is evidence in literature for small nonzero $\delta_{b}$ in this system 25 hence some simulations used $\delta_{b}\neq 0$.

**Table 1:** Model Parameters Used in Simulation Experiments

	Type	T_1,a_ (ms)	T_1,b_ (ms)	T_2,a_ (ms)	T_2,b_ (ms)	k_a_ (s^−1^)	*f*
Myelin water exchange	BM	1000	500	100	20	2	0.2
White matter MT	MT	779	779	45	12 × 10^−3^ [Link]	4.3	0.117
Caudate nucleus MT	MT	1087	1087	59	12 × 10^−3^ [Link]	2.3	0.061

By convention, compartment *a* is larger and *f* is the fractional size of compartment *b*. k_a_ is the exchange rate from *a* to *b*. The white matter and caudate nucleus MT models use parameters estimated for 1.5 T by Gloor et al (Table 1, ref. 26).

Magnetization transfer experiments used a super‐Lorentzian absorption lineshape model with T_2,b_ = 12 μs as described in the text, giving G(0) = 15.1 μs.

The white matter and caudate nucleus MT models are taken from measurements made at 1.5 T by Gloor et al (Table 1, ref. 26). The super‐Lorentzian lineshape function has been used for $G\left(\omega_{z}\right)$; as in 26, the function was extrapolated between ±1 kHz by fitting a spline to avoid the singularity at zero frequency. Systems with MT effects have been shown to exhibit bi‐exponential evolution of longitudinal magnetization 27, 28 with relaxation rates given by the two eigenvalues of $\Lambda_{L}$. The T_1_ observed from typical inversion recovery measurements (denoted as $T_{1}^{\text{obs}}$) is derived from the smaller (less negative) eigenvalue 27 as

(15) $$T_{1}^{obs}={\left\{\frac{R_{1,a}+k_{a}+R_{1,b}+k_{b}}{2}-\frac{\sqrt{{\left(R_{1,a}+k_{a}+R_{1,b}+k_{b}\right)}^{2}-4\left(R_{1,a}\;R_{1,b}+R_{1,a}k_{b}+R_{1,b}k_{a}\right)}}{2}\right\}}^{-1}.$$

For the white matter MT model, $T_{1}^{\text{obs}}$ = 779 ms.

All numerical simulations and analyses were performed using MATLAB R2015a (The MathWorks, Natick, MA, USA). A fully functional implementation is available to download at http://www.github.com/mriphysics/EPG-X . Code and experimental data for generation of all results presented in this paper are included (hash 4e8ba3d was the version at time of submission).

### Test 1: Steady State and Transient Behavior of Gradient‐Echo Sequences

Steady‐state expressions for spoiled gradient‐echo (SPGR) and balanced steady‐state free precession (bSSFP) sequences have been derived for BM and MT models elsewhere in literature 22, 26, 29, 30. The Appendix gives these solutions in terms of the matrices already introduced in the Theory section.

To validate the transient behavior, isochromat‐based simulations were also performed by solving the BM equations directly for a range of dephasing angles ψ, then integrating over the interval ψ∈[−π,π]. Simulations were performed using varying numbers of isochromats (N_iso_) spaced evenly over range [−π,π].

In this test, the EPG‐X(MT) simulations used the white matter MT model and EPG‐X(BM) simulations used the myelin‐water exchange model (Table 1). For comparison, single‐component (classic EPG) calculations were performed using T_1_ = 799 ms and T_2_ = 45 ms. Sequences with repetition time (TR) of 5 ms and flip angle (α) of 10° were simulated for 5 × T_1_, after which a steady state was assumed to have formed. For SPGR, simulations were repeated for different values of the quadratic RF spoiling phase increment $\Phi_{0}$ from 0° to 180°. $\bar{W}$ was computed by assuming hard pulses with maximum amplitude 13.5  μT.

Equation (1) implies that $M_{+}\left(\psi \right)$ and $M_{z}\left(\psi \right)$ may be obtained from the EPG predictions by performing an inverse fast Fourier transform (FFT) over “order” parameter *n* 31. For bSSFP, because net gradient area is zero, the familiar off‐resonance sensitivity of the bSSFP method may be found by applying iFFT to EPG predictions. These were compared with the steady‐state solutions of Equation (A2).

### Test 2: Multicomponent T_2_ Analysis of CPMG Data

The multicomponent analysis of multi‐echo CPMG spin‐echo data is a well‐known method for estimation of myelin fraction in white matter 32. If perfect 180° refocusing pulses are assumed, the multi‐echo signal can be analyzed using nonnegative least squares (NNLS) fitting to an exponential model 33. It is recognized that $B_{1}^{+}$ inhomogeneity will introduce other stimulated echoes that make the data deviate from this simple model 16. To explore any additional effects from exchange, we used EPG‐X(BM) simulations with the myelin‐water exchange model (Table 1) to simulate multi‐echo data for a range of exchange rates, $B_{1}^{+}$ scaling factors, and offset frequencies $\delta_{b}$. In each case, the simulated data were analyzed using the classic NNLS fitting approach, from which the estimated small pool fraction was taken as the area of smaller peak in the T_2_ spectrum.

Simulations used 50 echoes with 5 ms spacing. k_a_ was varied from 0 to 2.5 s^−1^ (for *f* = 0.2, this corresponds to infinite $\tau_{b}$ (i.e., no exchange) down to 80 ms). $B_{1}^{+}$ scaling factors from 0.75 to 1.25 were included. $\delta_{b}\;$ in the range of ±128 Hz (± 1 ppm at 3 T) was investigated with fixed $B_{1}^{+}$ scaling factor of 1.0. The NNLS was performed using the MATLAB function lsqnonneg.

### Test 3: MT in Transient Gradient‐Echo Sequences

Magnetization transfer effects have been shown to strongly affect the SPGR signal in the steady state 27, 29. Transient gradient‐echo sequences with variable flip angle, often following inversion pulses, have been used for MRF 21, 34; hence, we simulated a simple example of such a sequence to predict transient behavior. The sequence used an adiabatic inversion pulse followed by a series of 256 low flip‐angle RF pulses whose amplitude was varied sinusoidally (shown in the Results section); some pulse amplitudes were zero to allow for magnetization recovery. The RF pulse energies were 433 ${\text{ms}\;\mu T}^{2}$ for the inversion and $54.3\;\times \;\alpha^{2}\;{\text{ms}\;\mu T}^{2}\;$ for the small flip‐angle pulses ( α is the flip angle, rad). A constant TR of 12 ms was used with constant gradient area in each TR period, even those with zero flip angles. The sequence was simulated with EPG‐X(MT) using the white matter MT model (Table 1). Both bSSFP and SPGR were simulated using the same timing.

For consistent comparison, single‐compartment EPG was simulated using $T_{1}^{\text{obs}}$ and T_2,a_, as these are the parameters that would be measured using standard inversion recovery and spin‐echo methods.

### Experimental Measurements

Experiments were performed on a Philips Achieva 3T MRI scanner (Best, Netherlands). Two phantoms were made from sample tubes: water doped with 0.1 mM MnCl_2_ (expected to have no MT effect), and crosslinked bovine serum albumin (BSA; Sigma‐Aldrich, Dorset, United Kingdom), which has been suggested as a good material for replicating MT in human tissue 35. A 10% BSA solution (by weight) was prepared in distilled water, before treatment with glutaraldehyde (Sigma‐Aldrich) as described in 35.

The samples were imaged using the SPGR sequence described previously (TR = 12 ms, echo time = 2.9 ms), with frequency encoding aligned with the longitudinal axis of the tubes and phase‐encoding switched off to directly record echo amplitudes. Experiments were repeated with RF spoiling phase increment $\Phi_{0}$ set to 150° (default) and 117°. Single‐compartment relaxation times and apparent diffusion coefficient (*D*) were measured for each phantom, using inversion‐recovery TSE ( $T_{1}^{\text{obs}}$), multi‐echo spin echo (T_2_), and diffusion‐weighted spin echo (*D*). For the water phantom $T_{1}^{\text{obs}}$ = 899 ± 5 ms, T_2_ = 92 ± 2 ms, and *D* = 2.35 ± 0.18 × 10^−3^ mm^2^s^−1^; for BSA $T_{1}^{\text{obs}}$ = 1290 ± 25 ms, T_2_ = 92 ± 5 ms, and *D* = 1.92 ± 0.29 × 10^−3^ mm^2^s^−1^. Separate long TR multi‐flip‐angle measurements were made to precisely measure the effective B_1_ amplitude and M_0_ (including receiver coil scaling) in these phantoms; these measurements were used to match the measured echo amplitudes as closely as possible to EPG simulations.

As will be shown later, it was found that diffusion effects needed to be taken into account to accurately model the SPGR sequence. Diffusion can readily be accounted for in the EPG framework 12. We experimented with extending this to multicompartment models by applying the same treatment independently to each compartment; validity of this approach is discussed later. For the results presented in this paper, diffusion effects were only included for those relating to Test 3.

### Test 4: MT Effects in Multislice TSE Imaging

Multislice TSE is sensitive to MT effects 36, 37, as from the point of view of a given slice location, the acquisition of the other slices may be viewed as repeated off‐resonant irradiation, leading to attenuation of signals from some tissues in multislice compared with single‐slice acquisitions. To investigate this effect, we acquired data from a single healthy adult male volunteer (age 25; written consent was obtained before enrollment) using a Philips 1.5T Ingenia MRI system. A series of multislice TSE images were acquired using refocusing flip angles 180° and 120° and from one to 15 slices (odd numbers only). All acquisitions used 25 echoes, interecho spacing = 7.7 ms, and TR = 5 s. Acquired resolution was 1.5 × 1.5 mm^2^, slice thickness = 2 mm, and slice spacing = 2.78 kHz for the 180° sequences, and 3.13 kHz for the 120° sequences (RF pulse details are given in Table 2). A gap of 4 mm was used to avoid cross‐talk and default “odd‐even” slice ordering was used. Images were analyzed by drawing regions of interest in white matter, caudate nucleus, and cerebrospinal fluid.

**Table 2:** Radiofrequency Pulse Properties for Test 4

Sequence	Excitation pulse		First refocusing pulse		All other refocusing pulses	
	Flip (°)	Energy (ms ${\mu T}^{2}$)	Flip (°)	Energy (ms ${\mu T}^{2}$)	Flip (°)	Energy (ms $\mu T^{2}$)
120° refocusing	90	36.7	160	189.4	120	106.5
180° refocusing	90	32.7	180	213.1	180	213.1

Two sequences were used: one with 180° refocusing pulses and one with 120° pulses (in which the first refocusing pulse which was set by default to 160°).

The sequences were modeled using EPG‐X(MT) for both white matter and caudate nucleus MT models listed in Table 1. Off‐resonant excitation of other slices can be modeled by trains of pulses with zero flip angle for compartment *a*, but with saturation still applying to compartment *b*. The simulations were done from the point of view of the slice at the center of the group, as this was present in all acquisitions; these were run for three TR periods to ensure equilibrium was reached.

## RESULTS

### Test 1: Comparison With Existing Steady‐State Gradient‐Echo Solutions

**Figure 2:**
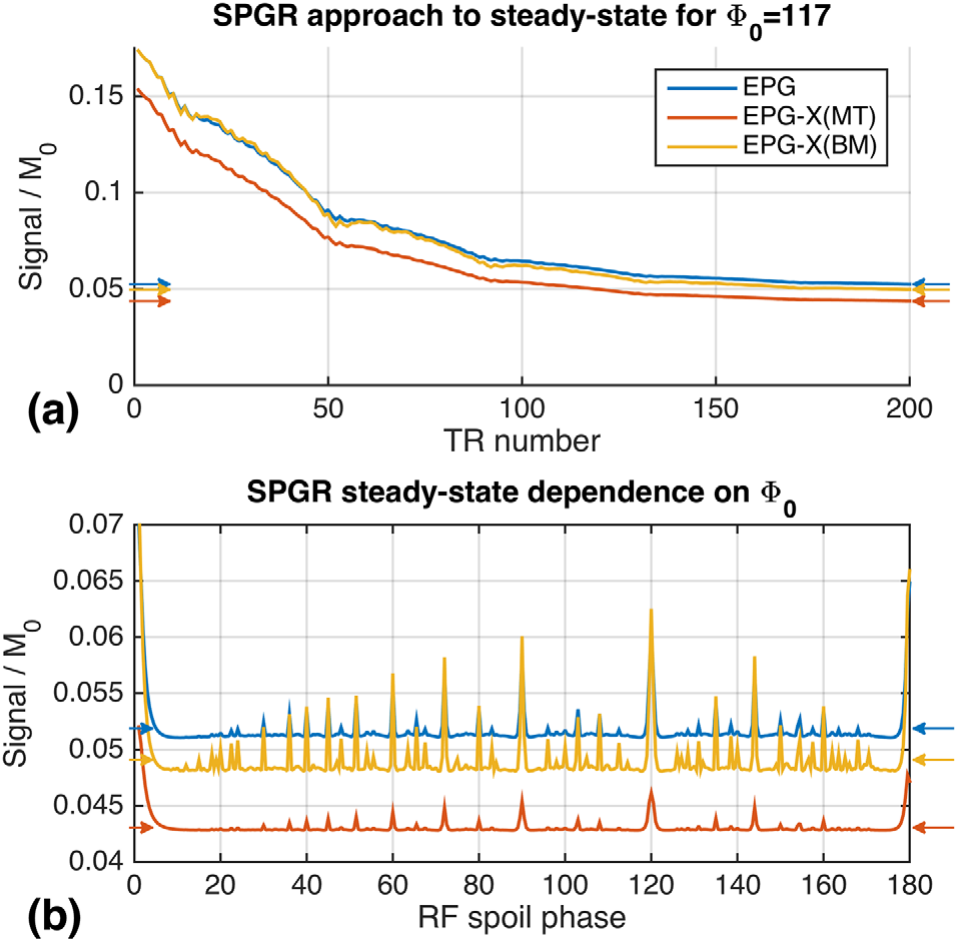
**a**: Approach to steady‐state for spoiled gradient‐echo (SPGR) sequence with repetition time = 5 ms, $\alpha =10^{\circ }$ computed with EPG, EPG‐X(BM), and EPG‐X(MT). Expected steady‐state signals marked with arrows were computed using Equation (A1). After approximately 200 repetition time periods, the different transient simulations each approach the expected steady state. **b**: Steady‐state signal as a function of RF spoiling phase increment $\Phi_{0}$. The steady‐state signals for the single‐compartment, MT, and BM models are all different. All are variable with $\Phi_{0}$, and as expected, do not always agree with the direct steady‐state calculations, which are computed assuming perfect spoiling.

Figure 2a shows the approach to steady state for SPGR with $\Phi_{0}=117{}^{\circ}$ for standard EPG and the proposed variants, compared with the ideal spoiling steady‐state values predicted by Equation (A1). All three curves approach the ideal spoiling steady state (arrows). Figure 2b shows the steady‐state values reached by the EPG methods for a range of $\Phi_{0}$ compared with the ideal spoiling prediction (as expected, strong variation with $\Phi_{0}$ is seen). https://onlinelibrary.wiley.com/action/downloadSupplement?doi=10.1002%2Fmrm.27040&attachmentId=208412584 explores this behavior further for the EPG‐X(BM) simulation by varying $\delta_{b}$ from 0 to 256 Hz (2 ppm at 3 T). For the values of $\Phi_{0}$ that result in good spoiling, not much variation with $\delta_{b}$ is seen; however, for the “spike” values such as 0° or 120°, an oscillatory dependence on $\delta_{b}$ is observed.

https://onlinelibrary.wiley.com/action/downloadSupplement?doi=10.1002%2Fmrm.27040&attachmentId=208412584 compares the predictions of the transient behavior for varying numbers of isochromats (N_iso_) with EPG and EPG‐X. Large discrepancies are seen for smaller N_iso_; however, for N_iso_ greater than or equal to the number of RF pulses, the two types of simulation agree exactly (differences ≈ 10^−15^), in line with 31.

**Figure 3:**
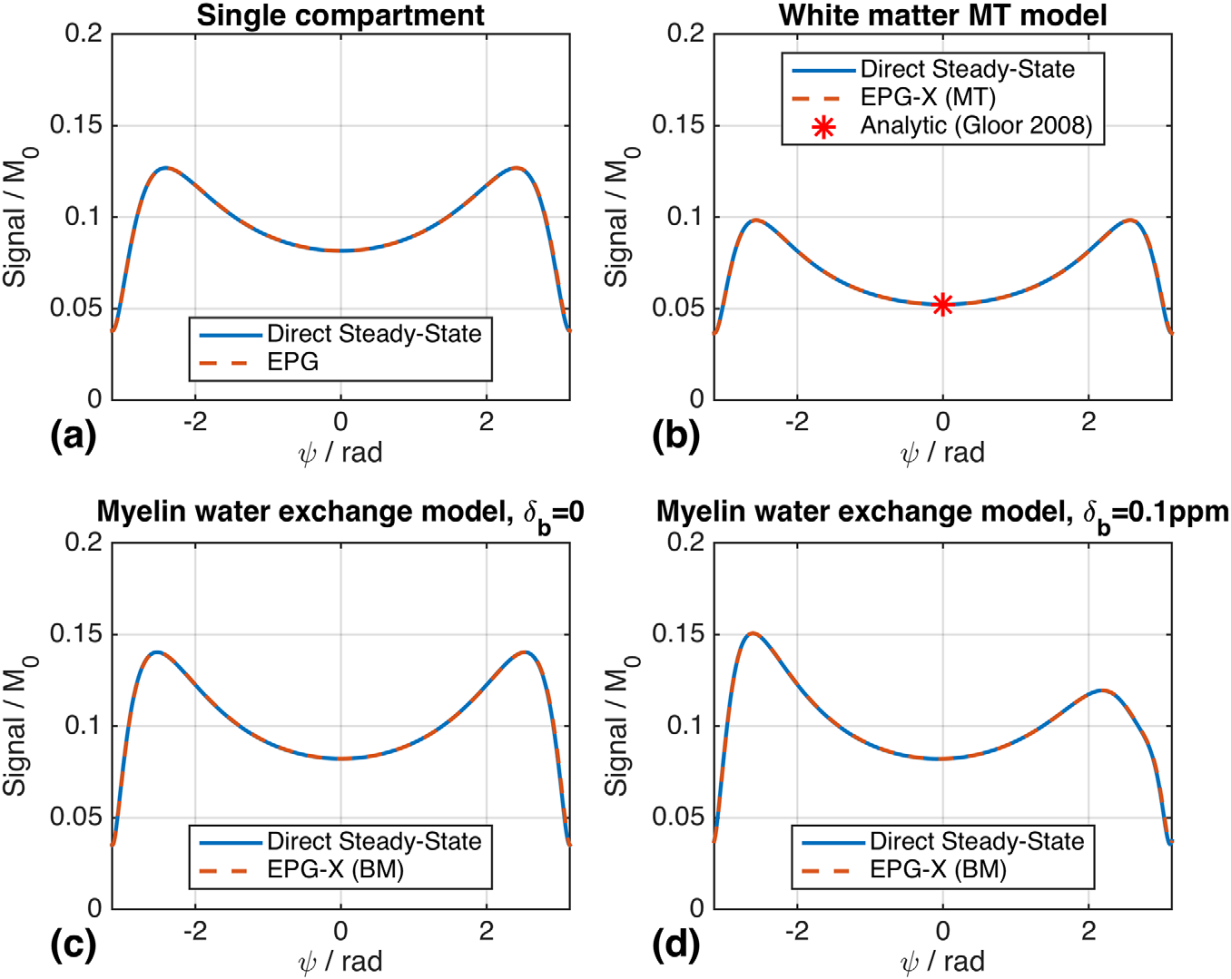
Balanced steady‐state free‐precision off‐resonance profiles computed using Equation (A2) compared with EPG/EPG‐X based predictions after reaching a steady state for (**a**) single‐compartment model, (**b**) white‐matter MT model, (**c**) myelin‐water exchange model with $\delta_{b}=0$, and (**d**) the same model with ${\;\delta }_{b}=12.8$ Hz (0.1 ppm at 3 T). The steady‐state solutions agree with the EPG/EPG‐X predictions for all cases. For EPG‐X(MT), there is also agreement with the analytic solution derived for ψ = 0 in 26.

Figure 3 compares the EPG and direct steady‐state solutions for bSSFP for all models; in these simulations, the myelin‐water exchange model was used twice, for $\delta_{b}=0$ and 12.8 Hz (0.1 ppm at 3 T). The profile for $\delta_{b}\neq 0$ (Fig. 3d) is markedly asymmetric. Each model produces quite different steady‐state behavior; however, in each case the agreement between EPG‐X and direct calculation is excellent, as is the agreement with the on‐resonance analytic expression for the MT case (red asterisk, ref. 26).

### Test 2: Multi‐echo CPMG Relaxometry

**Figure 4:**
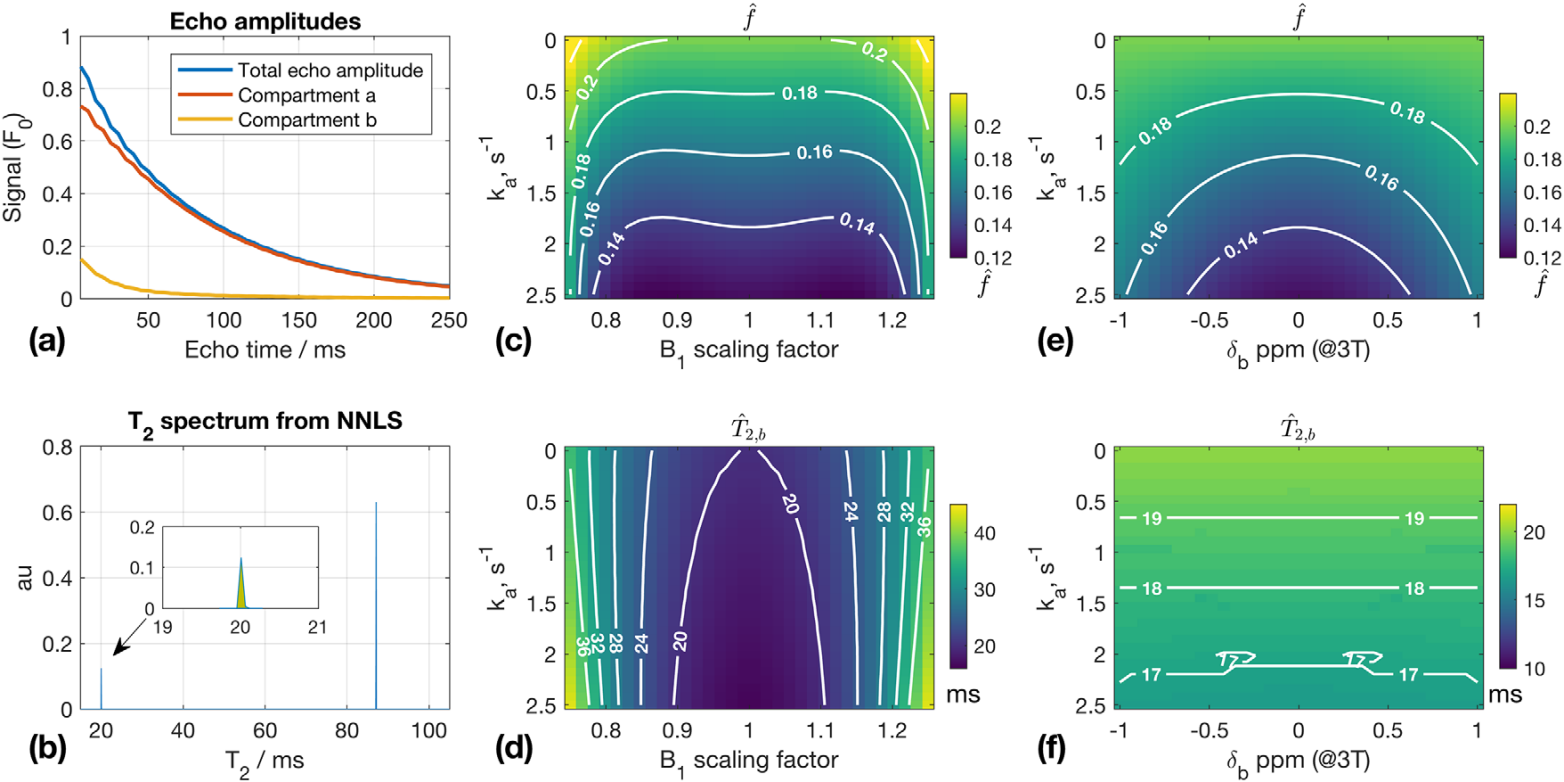
Results from Test 2. **a**: Example echo amplitudes for k_a_ = 2s^−1^ B_1_ scaling = 1.1; both compartments are shown, but only the total echo amplitude would be observed experimentally. **b**: T_2_ spectrum from data in (**a**) obtained using nonnegative least square (NNLS). Inset plot shows shorter T_2_ peak; ${\hat{T}}_{2,b}$ = 20.0 ms, $\hat{f}$ = 0.133 (obtained from peak area, shaded). **c, d**: $\hat{f}$ and ${\hat{T}}_{2,b}$ as functions of k_a_ and B_1_ scaling; $\hat{f}$ is primarily a function of k_a_. When k_a_ = 0, $\hat{f}$ is estimated correctly; otherwise, $\hat{f}$ tends to be systematically underestimated. ${\hat{T}}_{2,b}$ is primarily a function of B_1_ scaling. **e, f**: $\hat{f}$ and ${\hat{T}}_{2,b}$ as functions of k_a_ and $\delta_{b}$ ( $\hat{f}$ depends on $\delta_{b}$).

Figure 4a shows example echo amplitudes from the simulated multi‐echo CPMG data; Figure 4b has the corresponding T_2_ spectrum from the NNLS analysis. There are two peaks corresponding to the two compartments (T_2,a_ = 100 ms and T_2,b_ = 20 ms), and the fraction is estimated by taking the ratio of the peak areas (shaded in Fig. 4b); the NNLS estimated parameters are denoted as $\hat{f}$ and ${\hat{T}}_{2,b}$. Figures 4c and 4d show that although ${\hat{T}}_{2,b}$ varies strongly with B_1_ scaling, $\hat{f}$ is more strongly dependent on the exchange rate k_a_. For k_a_ = 2 s^−1^ (i.e., $\tau_{b}$ = 100 ms), Figure 4c indicates $\hat{f}=$ 0.133, a 33% underestimate. Figure 4e shows that $\hat{f}$ also varies weakly with $\delta_{b}$.

### Test 3: MRF‐Style Transient Gradient Echo

**Figure 5:**
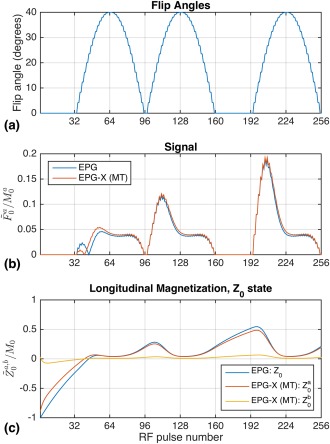
**a**: Variable flip‐angle train used for numerical and physical experiments. There are 16 different flip angles, including zero, which is included to allow some free recovery of magnetization. These pulses immediately follow an adiabatic inversion pulse (index 0). **b**: Predicted signals from white‐matter MT model (Table 1) and single‐pool comparison. The EPG‐X signals are normalized by (1‐f) to account for compartment *b* being “invisible.” MT leads to different behavior, particularly immediately after the inversion pulse. **c**: ${\tilde{Z}}_{0}^{}$ from the EPG and EPG‐X models. For EPG‐X, the level of saturation of compartment *b* changes dynamically, leading to altered dynamics of the observed signal when compared with a single‐compartment model.

The variable flip angle profile used is illustrated in Figure 5a. The figure also illustrates the expected signals (Fig. 5b) and evolution of longitudinal magnetization (unmodulated ${\tilde{Z}}_{0}^{}$ states (Fig. 5c)) in the white matter model for the SPGR sequence, comparing EPG‐X with single‐pool EPG using $T_{1}^{\text{obs}}$. The signal profiles are different, particularly immediately after the inversion (at the beginning of the sequence) when the magnetization in the MT system recovers more quickly. https://onlinelibrary.wiley.com/action/downloadSupplement?doi=10.1002%2Fmrm.27040&attachmentId=208412584 shows an equivalent result for bSSFP.

**Figure 6:**
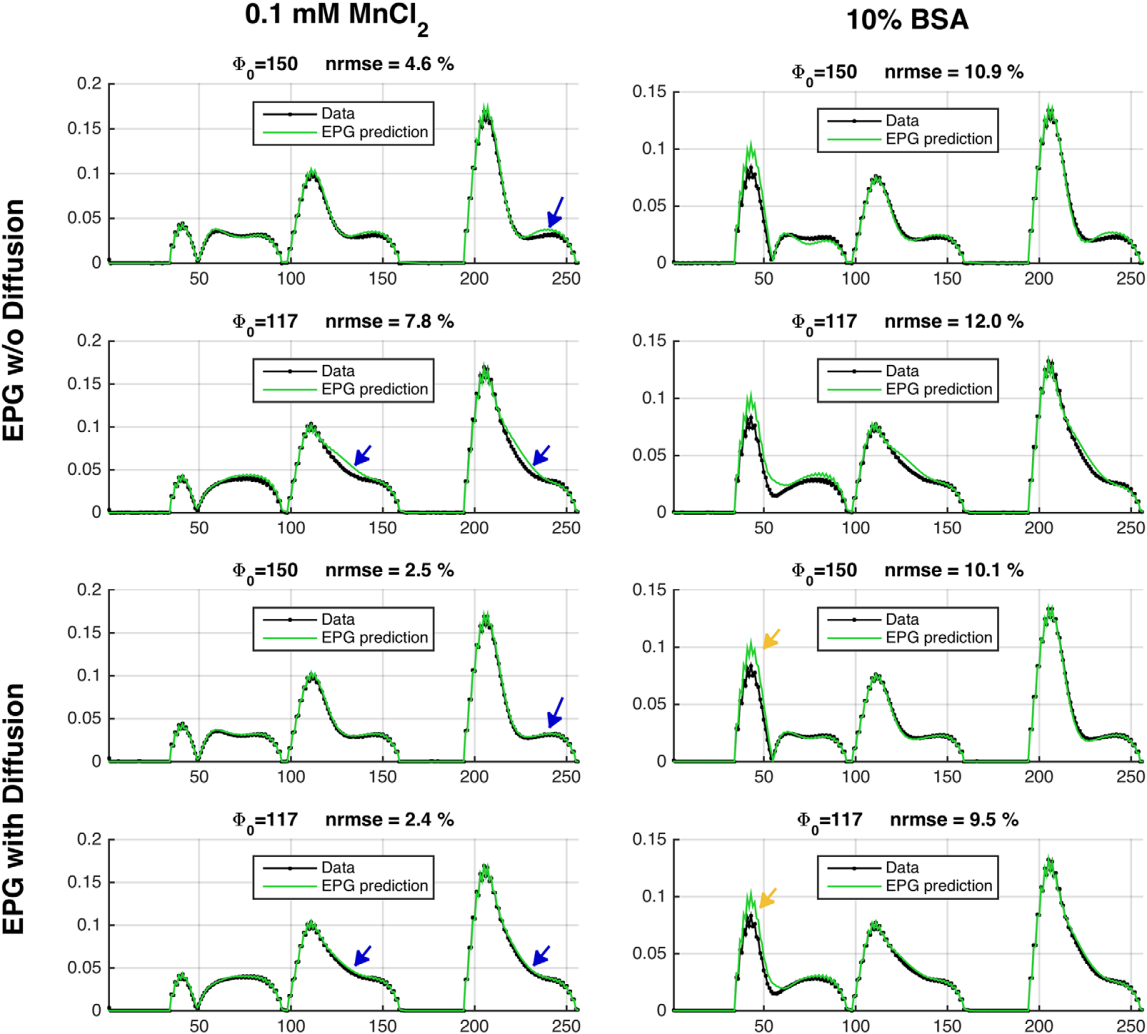
Experimental SPGR data compared with the single‐compartment EPG model. No fitting was performed (relaxation times, B_1_ scaling, and receiver/M_0_ scaling factors were experimentally measured). When diffusion is not included (top two rows), the match to EPG is not perfect (blue arrows). For the MnCl_2_ phantom, once diffusion is included (bottom two rows), the match is very good (normalized root mean square error (NRMSE) ∼2%). For the bovine serum albumin (BSA) phantom, there remain systematic differences (yellow arrows), suggesting that the single‐compartment model is not sufficient. Moreover, the observed signal profiles are quite different for the two different values of $\Phi_{0}$, as predicted by the EPG model.

**Figure 7:**
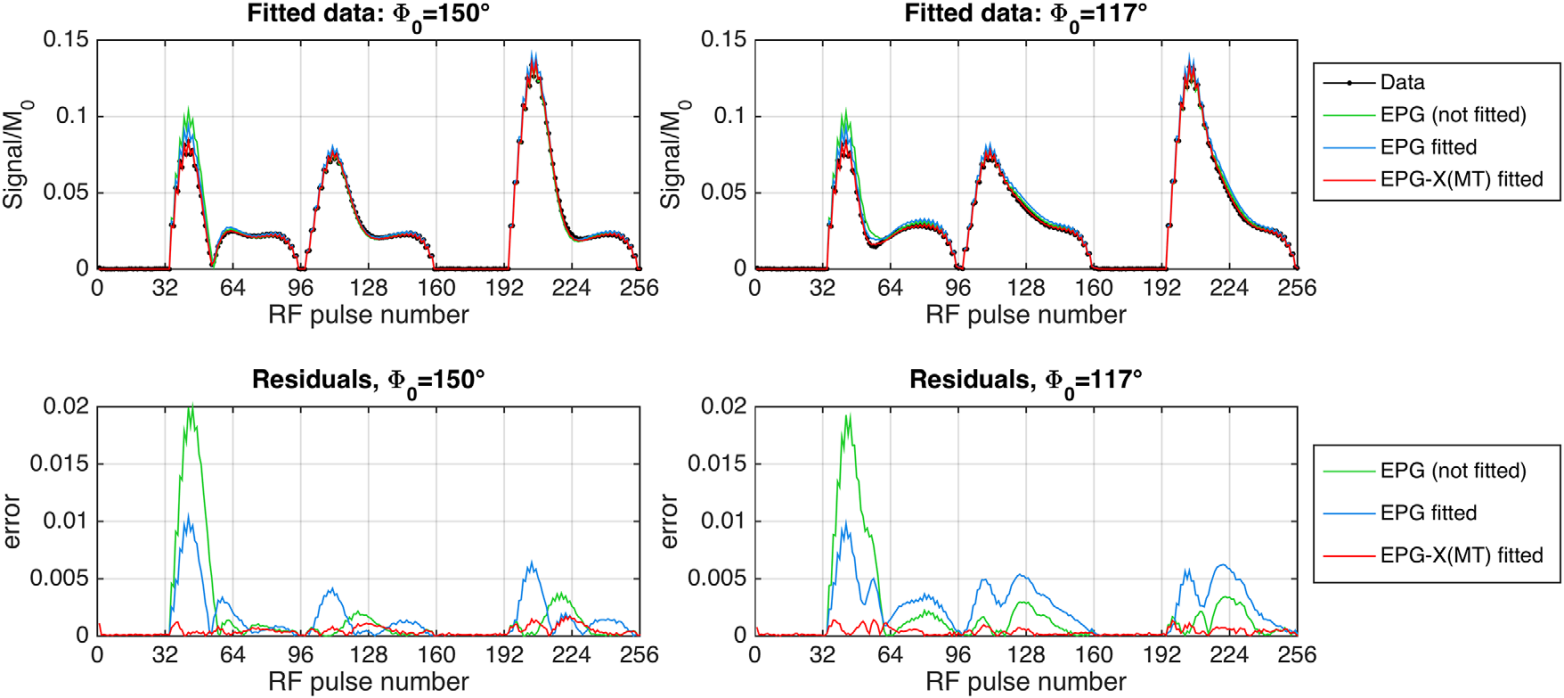
Result of fitting EPG and EPG‐X(MT) models to the BSA phantom data. Both values of $\Phi_{0}$ were fit simultaneously. Top row: Fits for both values of $\Phi_{0}$. Bottom row: Residuals. T_2_, *D*, and scaling factors were held fixed at experimentally measured values. For EPG‐X(MT), the k_a_, T_1,a_, T_1,b_, f, and G(0) were varied; the best‐fit parameters were k_a_ = 6.2 s^−1^, T_1,a_ = 1763 ms, T_1,b_ = 363 ms, G(0) = 29.4 µs, f = 0.100, which would yield $T_{1}^{\text{obs}}$ = 1283 ms. For single‐compartment EPG, the T_1_ was varied; the best fit was 1215 ms. The NRMSE before fit = 9.8%, single‐compartment EPG fit = 6.9%, EPG‐X = 1.3%.

Figure 6 compares experimentally the obtained SPGR data with standard EPG predictions; the data and model are not fitted together, and all necessary parameters and scaling coefficients were measured in calibration experiments. For the MnCl_2_ phantom, reasonable agreement is obtained using EPG; however, this is improved by including diffusion effects (blue arrows). Note also how the signal profiles from the two different RF spoiling phase increments $\Phi_{0}\;$ are quite different. For BSA, the match to single‐compartment EPG is slightly improved by adding diffusion effects; however, there are systematic differences particularly immediately after the inversion, similar to those observed in Figure 5 (yellow arrows). We hypothesized that these are caused by MT. The experimentally obtained data for BSA were fitted to EPG‐X(MT) predictions using least‐squares minimization, optimizing over *f*, T_1,a_, T_1,b_, k_a_, and G(0). Fitting used the MATLAB function fmincon including a nonlinear constraint enforcing consistency between estimated parameters and measured $T_{1}^{\text{obs}}$ (via Eq. (15)). Data for $\Phi_{0}=150^{\circ }\;\;$ and $\Phi_{0}=117^{\circ }$ were fit simultaneously. T_2,a_, D, and the overall scaling constant were fixed at the measured values. Diffusion was implemented in EPG‐X using the same approach as for standard EPG; the validity of this approach will be discussed later. Figure 7 shows the fit that could be obtained; there is very good agreement (root mean square deviation of 1.3%) using k_a_ = 6.2 s^−1^, T_1,a_ = 1763 ms, T_1,b_ = 363 ms, G(0) = 29.4 µs, f = 0.100. This combination would yield $T_{1}^{\text{obs}}$ = 1283 ms, which is consistent with the inversion recovery measurement (1290 ± 25 ms). As a control, the single‐compartment EPG model was also fitted to the data by varying T_1_. In this case, the best fit was T_1_ = 1215 ms, but significant residuals remain (Fig. 7; root mean square deviation of 6.9%).

### Test 4: Multislice TSE Imaging

**Figure 8:**
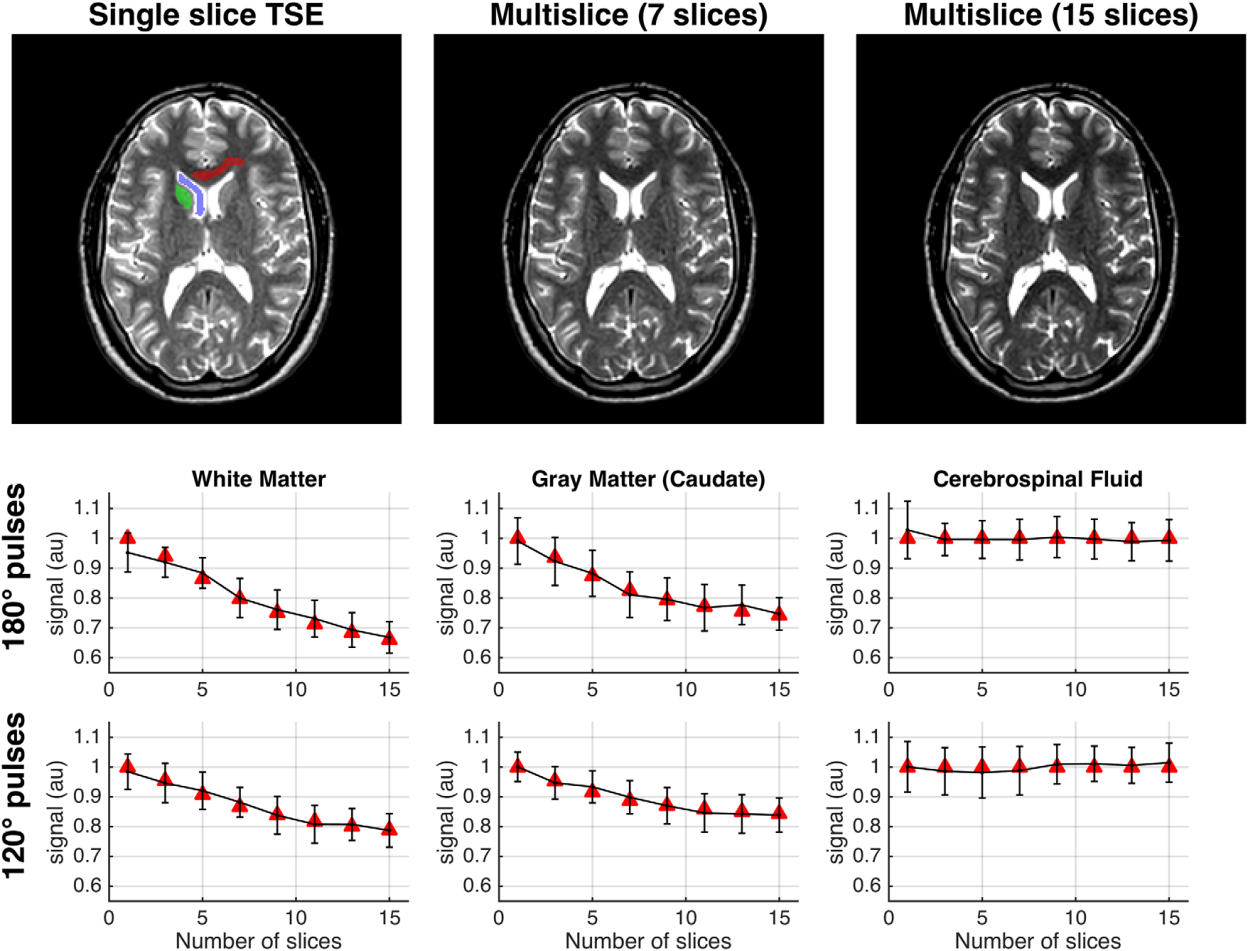
Top row: Turbo spin echo (TSE) images acquired using 180° refocusing pulses with single slice versus multislice, with 7 and 15 slices. The contrast changes as the number of slices increases, with the signal from white matter clearly falling. Lower rows: Predicted signals from EPG‐X(MT) model (red triangles) compared with the measured signals (error bars) from regions of interest indicated on the first image (red = white matter, green = caudate nucleus, blue = cerebrospinal fluid) as the number of acquired slices is changed. The upper row of plots shows data for 180° refocusing pulse sequence; the lower row is for 120° refocusing pulses. The signal from white matter is strongly attenuated, particularly with the 180° pulse sequence. Caudate nucleus shows similar, but slightly less severe, attenuation. The EPG‐X(MT) predictions match the experimental data within error. As expected, cerebrospinal fluid shows no attenuation, indicating no direct slice cross‐talk.

Figure 8 shows single‐slice and multislice TSE images from a healthy volunteer, using 180° refocusing pulses. As expected, white matter signal clearly decreases as the number of slices increases. The region‐of‐interest analysis (plots) compares the image data (error bars) with EPG‐X(MT) predictions (red triangles) for white matter, caudate nucleus, and cerebrospinal fluid. Data were scaled to compare with EPG‐X predictions by normalizing the mean signal over all experiments for each region of interest to the equivalent mean value from the EPG‐X predictions. The data and predictions match to within experimental error in all cases. The EPG‐X model correctly predicts the trends seen in white matter and caudate nucleus (the former having a stronger effect) and the effect of changing from 180° to 120° refocusing pulses. As expected, no change was observed in the cerebrospinal fluid signal (no MT effect), indicating that direct cross‐talk between slices is negligible.

## DISCUSSION

This work has introduced a general framework for the modeling of processes governed by the BM equations or their modified form for MT, using EPG. Essentially, the different compartments are described by separate phase graphs, which exchange with each other during evolution periods. For the MT case, the bound protons have no transverse components, so their phase graph contains only longitudinal states. The new model, referred to as the EPG‐X, has been validated by comparing directly with isochromat‐based simulations, and against existing steady‐state solutions. In addition to numerical validation, the EPG‐X(MT) model was found to agree well with in vivo measurements of signal attenuation in multislice TSE, and provided a plausible fit to transient gradient‐echo signal measurements using crosslinked bovine serum albumin (an MT phantom).

No fundamentally new biophysical model has been proposed in this work; rather, we have shown how existing methods can be incorporated into the EPG framework. As https://onlinelibrary.wiley.com/action/downloadSupplement?doi=10.1002%2Fmrm.27040&attachmentId=208412584 shows, EPG‐X methods produce identical results to direct isochromat‐based integration of BM equations, as long as the latter approach is sufficiently sampled 31. The EPG method is well‐established as a computationally efficient and intuitive approach to modeling MRI sequences, particularly those with multiple echo pathways such as TSE (see 3 for a detailed discussion). The EPG‐X extensions seek to retain these advantages for the modeling of more complex multicompartment systems. Calculations retain the same structure as the original EPG approach, allowing the use of established methods for truncating and hence accelerating EPG‐X calculations 10, 38. In addition to efficiency, the major advantage of the EPG approach is that it allows for intuitive analysis of sequences in which one or more echo pathways are isolated for measurement (TSE sequences and the many variants such as “hyperechoes” 6 are the classic example, MRF using an echo splitting technique 39 is a more recent application). In all of these cases, the proposed EPG‐X framework would allow for the effect of exchange or MT to be considered, and this may have a significant effect on predictions.

The examples in this paper focused on two different types of models, both of which are commonly used for quantitative MRI in the brain. The myelin‐water exchange model is used for steady‐state approaches like multicomponent DESPOT 22 and multi‐echo CPMG relaxometry 24, although for the latter, exchange between compartments is usually neglected. Typically, two significant peaks are observed in white‐matter T_2_ spectra from multi‐echo CPMG data 32, with the faster relaxing component (T_2_ ∼ 20 ms) identified as myelin water; the fractional size of this component is used to estimate the myelin‐water fraction. More recent work has found large variability in apparent myelin‐water fraction from tracts with similar myelin content in rat spinal cord 40, with differing levels of exchange suspected as a potential reason. Results from Test 2 (Fig. 4) suggest that exchange can lead to significant underestimation of the myelin‐water fraction, and hence might be able to explain these experimental observations. Therefore, EPG‐X could potentially be used for incorporating estimation of exchange parameters into multi‐echo relaxometry.

We also investigated the effect of compartment *b* frequency offset $\delta_{b}$; this is relevant to white‐matter imaging in particular, as many studies have shown that there is a nonzero mean frequency offset resulting from local susceptibility effects, which is also orientation dependent 25, 41. https://onlinelibrary.wiley.com/action/downloadSupplement?doi=10.1002%2Fmrm.27040&attachmentId=208412584 shows that the effect on SPGR is relatively minor; however, surprisingly, Figure 4e shows that $\delta_{b}$ does interact with NNLS analysis and alters the estimated myelin‐water fraction for CPMG data. For the very small frequency shifts expected (<0.1 ppm 41), however, this would lead to only small biases. Results from Test 1 concerning bSSFP (Fig. 3d) suggest that a frequency shift of this size would lead to an asymmetric off‐resonance profile. This result is very similar to measurements made in white matter by Miller et al 42; the proposed framework would allow for further investigation, including exchange. Similarly, recent work on chemical exchange saturation transfer (CEST) 47 has proposed using observed asymmetries in the bSSFP frequency profile as an alternative detection mechanism to more standard z‐spectrum saturation measurement. The latter approach to CEST measurement usually requires long saturation pulses; EPG is not useful for simulation of this interaction, as it is designed to characterize sequences rather than individual RF pulses. However the “sequence level” CEST detection method proposed in 47 can be simulated with EPG‐X(BM), and further work could use the framework to investigate CEST effects for other pulsed sequences.

The second type of model considered in this work focused on MT effects. For MRF, a simulation using white‐matter parameters (Fig. 5 and https://onlinelibrary.wiley.com/action/downloadSupplement?doi=10.1002%2Fmrm.27040&attachmentId=208412584) shows that MT will lead to altered behavior, which is particularly pronounced after inversion because of bi‐exponential recovery, which has been observed in human white matter (e.g., 28). Measurements on doped water and BSA phantoms (Fig. 6) also suggest that although the former can be well modeled using standard EPG (good agreement is obtained with no fitting to the data), a large residual is observed in the period after the inversion pulse in the BSA. This type of deviation might be a source of bias in MRF; however, the manner in which this would affect the final result remains to be investigated. The fact that the observed “fingerprint” profiles are affected by MT could also imply that there is potential for quantitative MT characterization with this type of sequence. To demonstrate this, we used nonlinear fitting to estimate the MT specific parameters (Fig. 7) from the BSA data. The fitted parameter values are consistent with the inversion recovery–measured $T_{1}^{\text{obs}}$. Furthermore, the estimated *f* = 0.1 agrees with the fact that the phantom was prepared with 10% BSA by weight. Contrastingly, fitting the single‐pool EPG model gave a best‐fit T_1_ that is inconsistent with the inversion recovery, and still produces large residuals. The implication is that these data are not fully characterized by a single T_1_ value, which is consistent with observations made by others 28, 29. Our results indicate that the EPG‐X model can explain the observed data more accurately, producing numbers that are consistent with independent inversion‐recovery measurements. Future work could explore the use of EPG‐X as the basis for generation of dictionaries for MT measurement via MRF.

Although most of the preceding exchange‐related effects are quite subtle, Test 4 showed that significant effects attributable to MT (signal attenuation in multislice TSE) can also be accurately predicted by EPG‐X (Fig. 8). Previous work found that a semi‐empirical model 37 could predict this signal loss, but required experimental measurement of sequence and tissue‐dependent coefficients to do so. The present work made accurate predictions using only literature tissue parameters (Table 1, ref. 26) and scanner‐reported sequence parameters. Hence, the EPG‐X method could be useful for pulse‐sequence parameter optimization that accounts for MT effects; these are not insignificant, for example more than a 30% reduction in white‐matter signal was observed when moving from single slice to a 15‐slice multislice acquisition.

Multicompartment models seek to explain complex underlying biological systems, but the choice of model is key. White matter is a particularly complex tissue; in this paper, we used “exchange” (BM) and MT models that are both relevant to white matter. This reflects the range of models that currently exist in the literature; the choice of model depends on the type of sequence being modeled as well as the tissue. For multi‐echo T_2_ relaxometry, the BM model is most relevant, as it is seeks to measure multiple compartments with appreciable T_2_. To evaluate the effect of off‐resonant saturation in multislice imaging, the MT model is most relevant. More complex mixed models that include multiple BM and MT compartments 43, 44 have also been proposed; Liu et al proposed a general framework for such systems 45. In this work we focused on two compartment models, but in principle the same arguments used in 45 can be used to extend the EPG‐X formalism to more compartments as well.

### Implicit Assumptions and Limitations

The EPG‐X model assumes that the underlying magnetization in both compartments forms a spatial distribution at a subvoxel‐length scale. Exchange couples Fourier configurations in one compartment with the same configuration in the other compartment, which is equivalent to assuming that exchange interactions couple these distributions locally (i.e., the magnetization in compartment *a* at one location couples with compartment *b* at the same location, but not adjacent locations). Isochromat‐based modeling methods (e.g., 45, 46) make the same assumption in the spatial domain. This is physically reasonable, as both chemical exchange and MT occur at the level of individual molecules, and compartmental exchange occurs over diffusion‐length scales, smaller than required for modeling the subvoxel magnetization distribution at spatial resolutions relevant to MRI.

Our data showed that diffusion effects must be accounted for to accurately match observed signals to EPG models. As far as we are aware, there is no commonly adopted equivalent model for the multicompartment case, as we are effectively combining the Bloch‐McConnell and Bloch‐Torrey equations. For the measured BSA data (Figs. 6 and 7), we took the most basic approach, which was to treat diffusion effects independently for both compartments but with the same diffusion coefficient for each. It might be expected that actually diffusion coefficients would be quite different for each compartment, and this could readily be achieved within the same framework. Further work is needed to identify the most appropriate biophysical model.

Finally, this work has been presented in terms of the “regular time increment” version of the EPG framework, which allows configuration states to be considered in terms of integer indexes only. Sequences with variable gradient directions and/or nonuniform timing are described instead using a continuous Fourier transform (see 3 for a detailed discussion). The theory put forward in this paper would generalize readily to this approach, as none of the exchange‐related operators are explicit functions of space.

## CONCLUSIONS

Extensions to the EPG framework to systems governed by the BM equations and modified forms for pulsed MT have been proposed. The new formalism named EPG‐X may be used to efficiently model a wide range of pulse sequences, and results indicate that for steady‐state sequences EPG‐X gives equivalent predictions to commonly used solutions. The EPG‐X model could prove to be useful for quantitative imaging, particularly for non‐steady‐state sequences in which accurate modeling of the transient response is necessary.

## Supporting information


**Fig. S1.** Steady‐state SPGR signal for myelin‐water exchange model as a function of $\Phi_{0}$ and $\delta_{b}$ (compare with Fig. 2b). As resonance offset $\delta_{b}$ is varied, no change in signal is observed for values of $\Phi_{0}$ that yield good spoiling (i.e., flat signal that is close to the desired ideal spoiling steady‐state value). However, for the spike values such as $\Phi_{0}=120$ ^°^ or 90^°^, the steady‐state signal oscillates as a function of $\delta_{b}$.
**Fig. S2.** The EPG and EPG‐X predictions plotted against isochromat predictions for SPGR approach to steady state with repetition time = 5 ms, flip angle = 10^°^. Isochromat ensemble simulations were repeated with increasing numbers of isochromats N_iso_ (from 10 to 1000); signal prediction comes from averaging the transverse magnetization M_+_ over the whole ensemble. **a, c, e**: The EPG and EPG‐X predictions (solid black line) are compared with isochromat simulations using differing N_iso._ Each different colored line is a different N_iso_; most prominent are blue = 10, rust = 30, and yellow = 50. **b, d, f**: Root mean square deviation between EPG and isochromat simulation for each case. The root mean square deviation drops as N_iso_ is increased and suddenly falls to approximately 10^−15^ for $N_{\text{iso}}\geq 200\;$ (number of RF pulses). At this point, EPG and isochromat predictions are effectively identical.
**Fig. S3. a**: Predicted signal as a function of pulse number and ψ for balanced steady‐state free precision. **b**: Profiles for ψ=0 and ψ=π2 (see dotted lines in (**a**)). The oscillations in the  ψ=0 case are caused by the “stepped” nature of the changing flip angles (see Fig. 5a). The effect of MT alters the signal dynamically, particularly after the inversion as with SPGR (Fig. 5). The difference between EPG and EPG‐X also changes as a function of off‐resonance parameter ψ. **c**: $\tilde{Z}$_0_ profiles; for EPG‐X, the saturation of compartment *b* varies dynamically.

## STEADY‐STATE SOLUTIONS FOR GRADIENT‐ECHO SEQUENCES

### Spoiled Gradient Echo

For an “ideally spoiled” sequence, we consider only a steady state formed by longitudinal components. The measured signal is given by

(A1) $$M_{+}^{ss}=\Sigma {\left\{I-\Xi_{L}\Theta \right\}}^{-1}\left\{\Xi_{L}-I\right\}\Lambda_{L}^{-1}C$$

where time increment Δt=TR is used in the definition of $\Xi_{L}$. Σ represents the excitation and sampling of longitudinal magnetization, and is defined differently for the BM and MT scenarios:

$$\begin{array}{l} \text{BM}\;\;\text{case}:\;\;\;\Sigma =[\sin\alpha \;\sin\alpha ]\\ \text{MT}\;\;\text{case}:\;\;\;\Sigma =[\sin\alpha \;0] \end{array}$$

In the BM case, the longitudinal components are summed to give total signal; for MT, the second component (taken to be the bound pool) does not contribute. The RF pulse transformation matrix Θ  is also defined differently for each case as follows:

$$\text{BM}\;\text{case}:\;\Theta =\left[\begin{array}{cc} cos\alpha & 0\\ 0 & cos\alpha \end{array}\right]$$

$$\text{MT}\;\text{case}:\;\Theta =\left[\begin{array}{cc} cos\alpha & 0\\ 0 & e^{-\bar{W\left(\omega_{z}\right)}\tau_{rf}} \end{array}\right]$$

Equation (A1) is a generalization of the Ernst formula for a single‐pool system.

### Balanced SSFP

For bSSFP, a coherent steady state among all components is considered. For the BM case written out for $M={\left[M_{+}^{a}\;M_{-}^{a}\;M_{+}^{b}\;M_{-}^{b}\;M_{z}^{a}\;M_{z}^{b}\right]}^{T}$, the steady‐state signal is given by

(A2) $$\begin{array}{l} M_{+}^{ss}=\Sigma {\left\{\Delta -\Theta e^{A\;TR}\right\}}^{-1}\Theta \left\{e^{A\;TR}-I\right\}A^{-1}C\\ A=\left[\begin{array}{cc} \Lambda_{T}+\Omega & 0\\ 0 & \Lambda_{L} \end{array}\right]\;C={\left[0\;0\;0\;0\;R_{1,a}M_{0}^{a}\;R_{1,b}M_{0}^{b}\right]}^{T} \end{array}$$

Δ represents a rotation of 180° about the z‐axis for both pools (accounting for the phase alternation in bSSFP), and Σ=[1 0 1 0 0 0]. Θ is defined in the same way as **T** (Eq. (12)), except that the rows and columns are re‐ordered to group terms for transverse components first, and longitudinal components second.

For the MT case, the same expression is used, except the rows and columns corresponding to $M_{+}^{b}\;$ and $M_{-}^{b}$ are deleted in all matrices, as are all coupling terms relating to $M_{+}^{a}\;$ and $M_{-}^{a}.$ The system is a 4 × 4 matrix, the modified RF pulse matrix (Eq. (14)) is used, $C={\left[0\;0\;R_{1,a}M_{0}^{a}R_{1,b}M_{0}^{b}\right]}^{T}$, and Σ=[1 0 0 0].
