# Supplementary material for: Extended phase graph formalism for systems with magnetization transfer and exchange
Source: Magn Reson Med. 2017 Dec 15;80(2):767–79. doi: 10.1002/mrm.27040 (PMC5947218; doi:10.1002/mrm.27040)
Supplement: Supplementary file 1 — Fig. S1. Steady‐state SPGR signal for myelin‐water exchange model as a function of Φ0 and δb (compare with Fig. 2b). As resonance offset δb is varied, no change in signal is observed for values of Φ0 that yield good spoiling (i.e., flat signal that is close to the desired ideal spoiling steady‐state value). However, for the spike values such as Φ0=120 ° or 90°, the steady‐state signal oscillates as a function of δb. Fig. S2. The EPG and EPG‐X predictions plotted against isochromat predictions for SPGR approach to steady state with repetition time = 5 ms, flip angle = 10°. Isochromat ensemble simulations were repeated with increasing numbers of isochromats Niso (from 10 to 1000); signal prediction comes from averaging the transverse magnetization M+ over the whole ensemble. a, c, e: The EPG and EPG‐X predictions (solid black line) are compared with isochromat simulations using differing Niso. Each different colored line is a different Niso; most prominent are blue = 10, rust = 30, and yellow = 50. b, d, f: Root mean square deviation between EPG and isochromat simulation for each case. The root mean square deviation drops as Niso is increased and suddenly falls to approximately 10−15 for Niso≥200 (number of RF pulses). At this point, EPG and isochromat predictions are effectively identical. Fig. S3. a: Predicted signal as a function of pulse number and ψ for balanced steady‐state free precision. b: Profiles for ψ=0 and ψ=π2 (see dotted lines in (a)). The oscillations in the ψ=0 case are caused by the “stepped” nature of the changing flip angles (see Fig. 5a). The effect of MT alters the signal dynamically, particularly after the inversion as with SPGR (Fig. 5). The difference between EPG and EPG‐X also changes as a function of off‐resonance parameter ψ. c: Z~ 0 profiles; for EPG‐X, the saturation of compartment b varies dynamically. [file MRM-80-767-s001.docx]

**Supporting Figures**

***
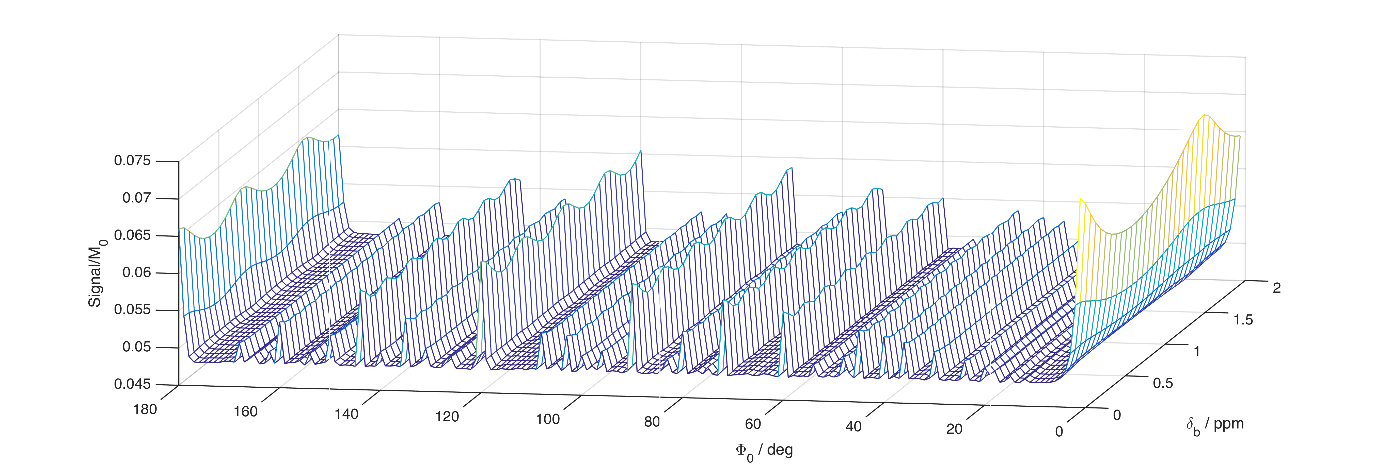
***

***Supporting Figure SF1:*** *Steady-state SPGR signal for myelin water exchange model as a function of* $\Phi_{0}$ *and* $\delta_{b}$ *(compare with Figure 2b). As resonance offset* $\delta_{b}$ *is varied, no change in signal is seen for values of* $\Phi_{0}$ *that yield good spoiling (i.e. flat signal that is close to the desired ‘ideal spoiling’ steady-state value). However for the ‘spike’ values such as* $\Phi_{0}=120^{\circ}$ *or 90° the steady-state signal oscillates as a function of* $\delta_{b}$*.*

*
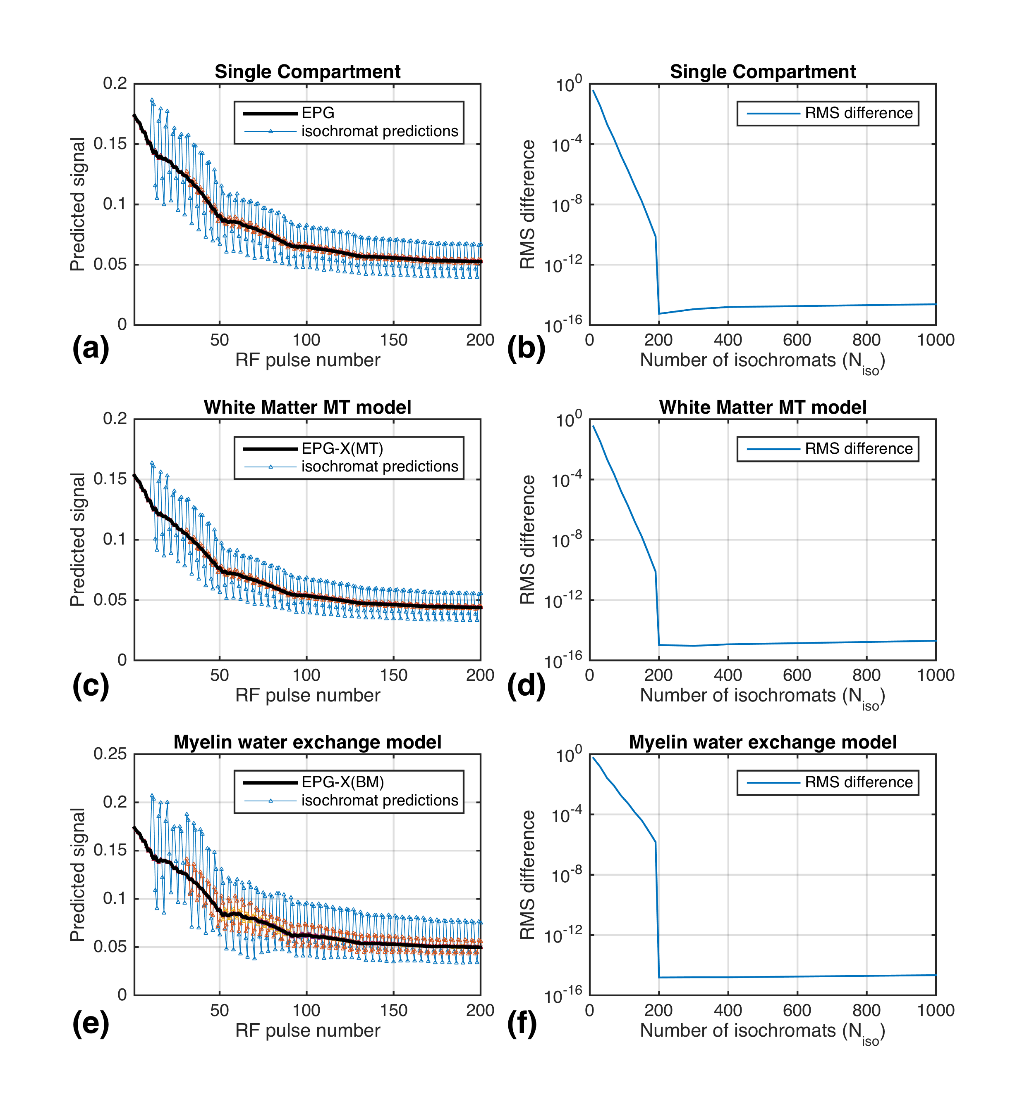
*

***Supporting Figure SF2:*** *EPG and EPG-X predictions plotted against isochromat predictions for SPGR approach to steady-state with TR=5ms, flip angle 10°. Isochromat ensemble simulations were repeated with increasing numbers of isochromats N_iso_ (from 10 to 1000); ‘signal’ prediction comes from averaging transverse magnetization M_+_ over the whole ensemble.* ***(a,c,e)*** *EPG and EPG-X predictions (solid black line) are compared with isochromat simulations using differing N_iso._ Each different coloured line is a different N_iso_; most prominent are blue=10, rust=30, yellow=50.* ***(b,d,f)*** *RMS deviation between EPG and isochromat simulation for each case. RMS deviation drops as N_iso_ is increased and suddenly falls to around 10^-15^ for* $N_{iso}\geq200$(number of RF pulses). *At this point EPG and isochromat predictions are effectively identical.*


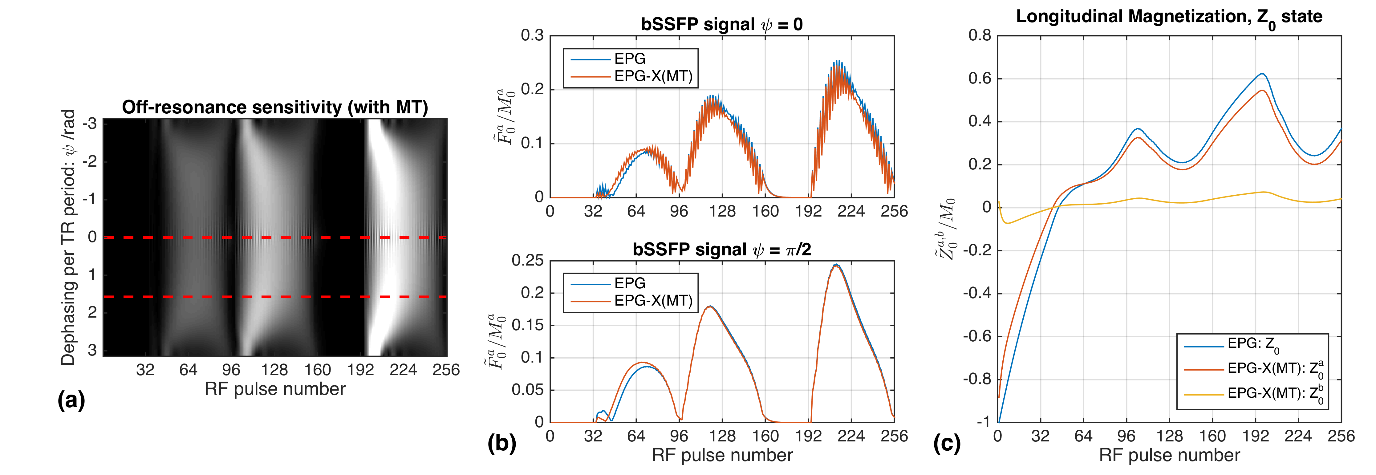


***Supporting Figure SF3: (a)*** *Signal as a function of TR number and ψ for bSSFP.* ***(b)*** *Profiles for* $\psi=0$ *and* $\psi=\frac{\pi}{2}$ *(see dotted lines on (a)). Note that the oscillations in the*$\psi=0$ *case are due to the ‘stepped’ nature of the changing flip angles (see Figure 5a). The effect of MT alters the signal dynamically, particularly after the inversion as with SPGR (Fig.5). The difference between EPG and EPG-X also changes as a function of off-resonance parameter ψ.* ***(c)*** $\tilde{Z}_{0}$*profiles; for EPG-X the saturation of compartment b varies dynamically.*
